# UK medical students’ attitudes towards their future careers and general practice: a cross-sectional survey and qualitative analysis of an Oxford cohort

**DOI:** 10.1186/s12909-018-1197-z

**Published:** 2018-07-04

**Authors:** Sarah Barber, Rachel Brettell, Rafael Perera-Salazar, Trisha Greenhalgh, Richard Harrington

**Affiliations:** 1 St Thomas’ Hospital, Westminster Bridge Rd, Lambeth, SE1 7EH London, England; 2 Nuffield Department of Primary Care Health Sciences, Radcliffe Primary Care Building, Radcliffe Observatory Quarter, Woodstock Rd, Oxford, OX2 6GG England; 3 Medical Statistics, Nuffield Department of Primary Care Health Sciences, Radcliffe Primary Care Building, Radcliffe Observatory Quarter, Woodstock Rd, Oxford, OX2 6GG England; 4 Primary Care Health Sciences, Nuffield Department of Primary Care Health Sciences, Radcliffe Primary Care Building, Radcliffe Observatory Quarter, Woodstock Rd, Oxford, OX2 6GG England; 5 Oxford graduate-entry medicine course, Medical Sciences Division, John Radcliffe Hospital, Level 7, Headington, Oxford, OX3 9DU England

**Keywords:** Career choice, Attitude, Medical schools, Medical students, General practitioners, General practice, Family practice, Primary health care, Physician shortage, Surveys and questionnaires

## Abstract

**Background:**

Against the background of the recruitment crisis in general practice, we aimed to determine what United Kingdom (UK) medical students value in their future careers, how they perceive careers in general practice (GP) and what influences them.

**Methods:**

Cross-sectional survey of 280 final and penultimate year medical students at the University of Oxford, with questions relating to career choices, factors of importance when choosing a career and attitudes towards general practice as a career. Quantitative methods included cluster analysis, chi squared tests of independence and logistic regression analysis. Qualitative data were analysed thematically using the Framework method.

**Results:**

Response rate was 89% (280/315). 40% of participants said that general practice was an attractive or very attractive career option. Respondents valued job satisfaction, work-life balance and close relationships with patients. However, fewer than 20% of respondents agreed that community-based working was important to them and many (often citing particular GPs they had observed) felt that general practice as currently structured may not be satisfying or fulfilling because of high workload, financial pressures and externally imposed directives. 63% perceived GPs to have lower status than hospital specialties and 49% thought the overall culture of their medical school had negatively influenced their views towards general practice. Some respondents considered that general practice would not be intellectually challenging or compatible with a research career; some appeared to have had limited exposure to academic primary care.

**Conclusions:**

With the caveat that this was a sample from a single medical school, medical students may be put off careers in general practice by three main things: low perceived value of community-based working and low status of general practice (linked to a prevailing medical school culture); observing the pressures under which GPs currently work; and lack of exposure to academic role models and primary care-based research opportunities. To improve recruitment of the next generation of GPs, medical schools must provide high quality placements in general practice, expose students to academic role models and highlight to policymakers the links between the current pressures in UK general practice and the recruitment crisis.

**Electronic supplementary material:**

The online version of this article (10.1186/s12909-018-1197-z) contains supplementary material, which is available to authorized users.

## Background

UK general practice is experiencing a crisis in recruitment and retention at a time of unprecedented demand. Health Education England has mandated that 50% of medical graduates should enter general practice [1] but no medical school is close to achieving this. This reflects a worldwide trend in which generalism is becoming less popular than specialism, with reports of shortages of GPs or family doctors in the United States (US) [2], Canada [3], Australia [4] and elsewhere.

Many reasons have been suggested for the recruitment crisis in general practice. Medical school factors can be thought of in relation to the three curricula defined by Hafferty: the formal, the informal and the hidden [5]. The formal curriculum may favour specialities through the disproportionate amount of time spent in hospital placements. This in turn limits exposure to the informal curriculum (ad-hoc, unscripted, interpersonal teaching) within primary care. Particular attention has been paid to the hidden curriculum: the “set of influences that function at the level of organisational structure and culture” [5]. The idea of “ending up” in primary care or being “just a GP” may stem from and be perpetuated by the subtle denigration (so-called “badmouthing”) of general practice that students and junior doctors encounter on hospital rotations [6–8]. A US study compared 20 medical schools and found that students attending schools with higher levels of “badmouthing” were less likely subsequently to practice primary care [9]. In the UK, certain medical schools have traditionally had low rates of progression to general practice [10] leading to accusations of “rank discrimination” against this career option [11].

External factors contributing to the recruitment crisis include the current pressures on primary care, linked to falling investment in real terms. Fewer than half of UK GPs report being satisfied with their job - the lowest proportion since 2001 [12]. Moreover, the appeal of hospital practice may have increased with the introduction of restrictions in working hours, at a cost to general practice recruitment [13, 14].

The UK Medical Schools Council has collaborated on two reports aiming to shed light on the complex medical school environment and frame solutions to the recruitment crisis. In 2016, “By Choice, Not by Chance” identified three key issues: “tribalism”, “negativism” and financing of undergraduate education [6]. In 2017, a grey literature report “Destination GP”, based on an online survey across several UK medical schools, highlighted the role of peers and role models, badmouthing and misinformation about primary care and the medico-political climate [15].

This study aimed to determine the views of medical students at Oxford University towards their future careers in general, and general practice in particular, in order to inform curriculum planning at Oxford and also contribute to the wider knowledge base.

## Methods

A cross-sectional study into the attitudes of Oxford medical students towards their future careers and general practice was conducted. Ethical approval for the study was obtained from University of Oxford Central University Research Ethics Committee (5/5/2016, R45420/RE001).

Based on findings in previous literature (see Background), a 19-item questionnaire (Additional file 1) was designed which combined dichotomous, multiple choice, demographic and Likert response scale questions with free text questions. Minor refinements were made after piloting the questionnaire with 4 individuals. In May 2016, an invitation to participate in the survey was sent to all the final and penultimate year students by email, alongside an introductory letter. At the time of receiving the questionnaire, all students would either be doing or have done an 8-week community block, which included 3 days a week in general practice for 6 weeks (year 5 GP placement), under the supervision of a single GP (year 5 GP tutor).

The survey was open for six weeks. Reminders were sent to non-responders and completion of the questionnaire was considered consent to participate in the study.

Research questions addressed in the survey centred around: career choices and influencing factors, socio-demographic factors, research ambitions and the attractiveness and status of general practice.

Quantitative data were analysed using R, SPSS and Excel. A cluster analysis in R was conducted to determine whether career interests showed some form of grouping. A chi-square test of independence was performed using Excel to examine the relationship between attractiveness of general practice as a potential career and gender, course type (standard 6-year course vs. graduate-entry 4-year course) and school type (non-selective state school in UK vs. selective state school in UK vs. private school in the UK vs. other). Further chi-squared tests were performed in Excel to determine the independence of the attractiveness of general practice and a) the perception of the professional status of GPs compared to hospital specialists, and b) the perceived overall culture of the medical school. A logistic regression analysis was completed in SPSS. We modelled the association between the attractiveness of a career in general practice (attractive or very attractive = 1) and a list of importance factors (Q4) and influencing factors (Q10). We performed separate models for each, one for influencing and one for importance factors and then combined those variables regarded as positively associated in a final model using a stepwise approach. In this way, we show that both the importance and influencing factors work independently, rather than competing as explanatory factors.

A thematic analysis of the qualitative data was undertaken using the Framework method [16], guided by a qualitative expert (TG). The free text data (which consisted of brief one- or two-sentence responses to open-ended questions) were transferred to a Word document and then pasted into Excel spreadsheets under a series of thematic headings. As the analysis developed, some headings were combined and others split into further sub-themes. In this way, the coding developed iteratively and became more refined as successive responses were added. We applied the theoretical notion of the hidden curriculum to inform the analysis.

## Results

### Overview of sample

Two hundred eighty students participated in the study, giving an overall response rate of 89%. 49% of participants were female; 85% were on the standard 6-year course (rather than the 4-year graduate-entry course). Responders did not differ significantly from the overall student population in these proportions (47% and 84% respectively). Over 30% of responders had attended non-selective state schools, 20% selective state schools and 43% private schools in the UK; 6% had attended secondary school outside of the UK, and 1% did not fit into these categories. 80% of responders provided some free-text responses (1066 comments in total), thus providing a very rich set of qualitative data.

### Quantitative data

**Fig. 1:**
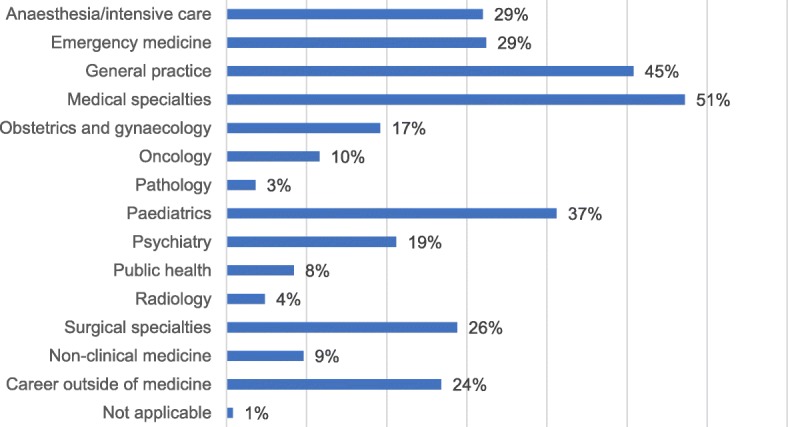
A bar chart to show the proportion of responders considering each career option (Q1, unlimited choice)

Figure 1 shows the proportion of responders considering each career option. The median number of career choices selected was 3, from a list of 15 (unlimited choice), with 45% of respondents indicating that they were considering a career in general practice. No clusters were found in these data. 70% of participants indicated that they started to consider their career options during clinical school (years 4–6). 51% of students had applied or were considering applying for a training post that included protected research time.

Ten percent of respondents rated general practice as very attractive as a potential career choice; 30% rated it as attractive, 28% as neutral, 24% as unattractive and 9% as very unattractive. These responses showed no significant relationship with gender (*p* = 0.10), type of course (*p* = 0.16) or school type (*p* = 0.12) in a chi squared test of independence.

When asked to compare the status of general practice to that of hospital specialists, 63% felt it was lower, 31% about the same, and 5% did not know. 0% said higher. There was no significant relationship between perceived attractiveness of general practice as a potential career and the perception of the professional status of GPs compared to hospital specialists (*p* = 0.25).

**Fig. 2:**
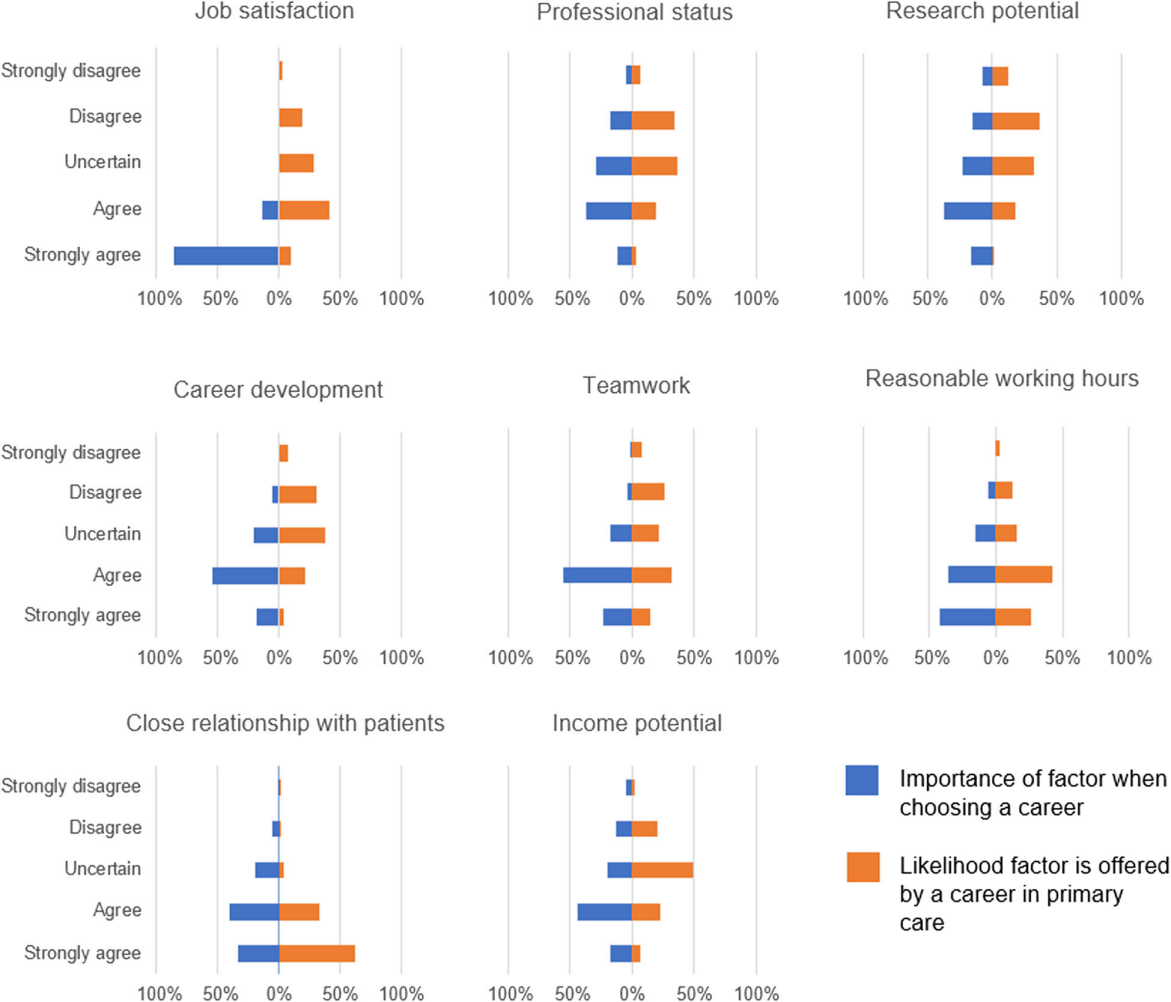
Bar charts comparing responses to Likert scale questions about the importance of a factor when choosing a career (Q4) and the likelihood that same factor would be offered by a career in primary care (Q9)

Participants were asked what factors they valued in a career. For some factors, this could be directly compared to the likelihood of a factor being offered by a career in general practice. This is shown in Fig. 2. Factors, ordered in importance from most to least, were as follows: job satisfaction (99% agree or strongly agree), teamwork (79%), flexibility in location (78%), reasonable working hours and close relationship with patients (74%), career development (72%), income potential (62%), research opportunities (55%), professional status (48%), length of training (44%) and community-based working (19%). Factors thought likely to be associated with a career in general practice were ordered in likelihood from most to least as follows: close relationship with patients (95% agree or strongly agree), reasonable working hours (69%), job satisfaction (49%), teamwork (46%), higher income (29%), career development (25%), professional status (23%) and research opportunities (19%). Notably, almost 50% of students were uncertain about income as a GP compared to hospital specialties.

**Fig. 3:**
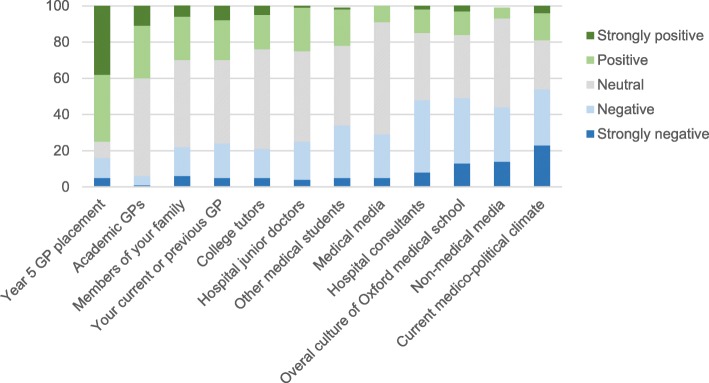
Stacked column chart showing the Likert scale responses to different influencing factors (Q10), ordered from most positive (left) to most negative (right)

Figure 3 shows the Likert scale responses to different influencing factors. The most positive influencing factor was the year 5 GP placement (average rating score 2.09 where strongly positive =1 and strongly negative = 5). The most negative influencing factor was the current medico-political climate (average rating score 3.53). No significant relationship between the attractiveness of general practice as a potential career and the perception of the overall culture of the medical school (*p* = 0.24) was found.

The logistic regression analysis found that the best fitting explanatory variables for the attractiveness of a career in general practice included in the final single model were the following factors: the year 5 GP placement, community-based working, reasonable working hours and the current medico-political climate. This final model predicted 73% of the responses correctly (Additional file 2).

### Qualitative data

A striking feature of the free text responses was that very few responders made exclusively negative statements. Most made a combination of positive and negative statements, often in the same sentence (e.g. *“If it was just the clinical aspects, GP would be perfect. However, the social / government nonsense is enough to put me off”* [S11]), suggesting that at this stage in their careers, students were still weighing up the pros and cons of general practice as a career option. It was also striking that whilst some perceptions expressed in the free text data could be attributed clearly to the formal medical school curriculum (e.g. “facts” that the students had learnt on their general practice placements), most of the free text statements were more closely aligned with the “hidden curriculum” of unconscious and incidental influences *beyond* what was formally or intentionally taught.

Three positive perceptions of general practice, which were widely but not universally held, were an expectation of good work-life balance, salary and/or job security, and a high degree of professional fulfilment and autonomy (*“Typically I imagine a better working environment and better hours and more control”* [S30]). Some respondents who expressed a strong preference for a career in hospital medicine or surgery described general practice as a good back-up plan (*“…I certainly would be open to converting to GP job at a later stage…”* [S186]).

Most negative statements about general practice could be divided into four over-arching categories that resonated strongly with the statistically significant findings from the quantitative data: a strong preference for a different career; perceptions about the current reality of general practice in the UK; perceptions about the generalist and professionally isolated nature of general practice; and perceived low status of general practice. We consider these in turn.

Some students had already developed a strong preference for a different career. They gave good personal reasons for this (e.g. the technical challenges of surgery or strong inspiration from a role model in a particular clinical specialty); these students rejected general practice primarily because their interests lay elsewhere. However, firm commitment to a specific career was uncommon in this cohort.

Many students felt that a potentially fulfilling and family-friendly career option, general practice, was currently in serious jeopardy because of long hours, high stress and numerous external directives that would reduce their sense of autonomy. These perceptions appeared to be the result of the student’s direct experience on their general practice attachment or (in some cases) observing a GP parent (*“The hours are brutal (14 hour days!) despite the idea of it being more ‘family friendly’…”* [S54]). Many students said changes in working conditions would make them more likely to consider general practice (“…[if] *there wasn’t a mountain of paperwork and we would get more than a few minutes with each patient…”* [S11]).

Respondents expressed a range of divergent views on general practice as a “generalism”. Some believed that generalism would provide professional fulfilment by exposing them to a wide variety of conditions and intellectual challenges (*“…I felt that the variety in work [types of patients/health needs] was attractive as well, especially the diagnostic challenge and juggling uncertainty and risk…”* [S136]). Others viewed generalism negatively, defining it in terms of minor and uninteresting ailments (*“Lots of necessary but tedious consultations...”* [S18]), with referral of [more interesting] complex cases to specialists (*“Don’t get to the bottom of problems – refer up the chain”* [S85]). This depiction of general practice as dealing with “easy” and mundane cases and passing the “difficult” and intellectually challenging ones to specialists contrasted with comments from other students who saw general practice as one of the most challenging careers both intellectually (*“…breadth of knowledge requires is very daunting”* [S81]) and logistically (*“Very difficult job, the idea of seeing a patient in 10 minutes seems overwhelming, and I would be worried I’d miss things and patients would feel rushed”* [S96]).

Some students appeared to associate general practice with professional isolation (*“I’d be worried it would be a bit lonely and unsupported”* [S20]) and with an absence of intellectual stimulation and research opportunities (*“Would like to do a PhD later in my career and GP would limit this”* [S69]). Interestingly, these statements were often couched in speculative terms and made from what appeared to be a position of ignorance (*“…there is less opportunity to be ‘academic’ as far as I know”* [S90]).

The culture of the medical school and attitudes of hospital doctors were often cited as negative influences on students’ perceptions of general practice *(“… ‘We’re not training you to become GPs’ was one quote in a lecture”* [S130] and *“Most medical students and Oxford Medical School/JR [John Radcliffe] consultants are rather dismissive about ‘GP land’”* [S136]). Some students criticised the badmouthing of GPs (*“Negative attitudes from secondary care professionals towards GPs are not uncommon. I dislike these attitudes which are often unfounded”* [S147]), but acknowledged its effect (“*I don’t consider them [GPs] inferior, but I feel inevitably its influence [sic] me”* [S249]). Most students reported that they had had a positive experience during their year 5 GP placement, and many named a particular GP (most commonly, their GP tutor) as a person who had particularly encouraged them to consider general practice as a career option (*“I have known a handful of GPs which I consider to be of the most inspiring people I’ve met in my medical training so far…”* [S32]). A few students named GP trainees as positive role models (*“…they show that young people do go into GP and are good advocates for the job”* [S20]). In sum, negative comments from specialists about general practice in general were offset (to some degree at least) by inspirational role models from *particular* GPs. Less commonly, students depicted a specific GP as uninspiring (*“My personal GP has been quite dismissive and unhelpful”* [S78]).

This overall picture was sometimes complicated, however, by comments from students who reported GPs (including their own GP tutor) directly discouraging them from considering primary care (*“Unfortunately every GP placement I have been on, my tutors have advised against it and said they do not enjoy their job”* [S200]) and by observing stress and burnout in particular GPs (*“…I cannot help but see how my father, who is a GP nearing the end of his career, has been jaded by significant changes to the job in the past ten years. He is an extremely positive person, but it seems clear that doctors like him have been abused lately…”* [S225]).

In sum, analysis of free text responses reveals that the students in our sample appeared to have been strongly influenced by both the formal curriculum (the clinical case load of general practice and exposure to a generalism) and a troubling *hidden* curriculum in which general practice was “badmouthed” by specialists and experienced as stressful, lacking autonomy and unfulfilling.

## Discussion

### Summary of principal findings

Forty percent of surveyed students considered general practice a potentially attractive career option. However, a subsequent survey of career destination of this cohort suggests that only 14% of them entered general practice [17]. This falls significantly short of the Health Education England goal of 50% of new graduates entering general practice.

Through logistic regression analysis we found four key explanatory variables to explain attractiveness of general practice, which are overlapping and link to wider themes. First, the value of community-based working. Clearly this is fundamental to general practice, so it is particularly concerning that less than one-fifth of students rate it as important in choosing a career and few students explicitly refer to community working in their free text responses. Second, the importance of reasonable working hours. A factor valued highly by respondents, and still associated with general practice by many. However, this is under threat from the third variable identified: the influence of the current medico-political climate. This was rated as the most negative influencing factor in the quantitative analysis. The free text responses further support our interpretation that students are fearful of the uncertain future for primary care within the National Health Service, and already have begun to see the erosion of its positive elements as they observe the pressures under which GPs currently work. The fourth variable was the year 5 GP placement. The quantitative results demonstrate the positive influence this was on the majority of students, with some GP tutors proving to be inspirational role models. However, the free text responses also reveal the extreme negative impact of some GPs who have directly warned students to seek careers in other specialties, or were visibly stressed and disillusioned at work.

Much of the motivation for this study was to explore the role of status and medical school culture. Neither emerged as key explanatory variables for the attractiveness of general practice. Nevertheless, there is indisputable evidence that students view GPs to have lower status than hospital consultants, and direct quotes in free texts confirm examples of this being reinforced within the teaching institution. Hospital consultants are seen as a more negative influence than either neutral or positive, with examples of badmouthing provided by the qualitative data. The role of medical students is less clear. As an influencing factor the majority of students described their peers as neutral. A handful of free text responses allude to a negative peer effect.

The low status of GPs may relate to the negative view of generalism held by some students; that is the belief that general practice work would be easy, dull and devoid of opportunities for career development or research (allegedly reinforced by negative stereotypes from hospital doctors). It appears that GPs are unable to quash this view, either because of limited interactions with students (academic GPs are a largely unknown entity to students) or due to low morale, desperation and burn out.

### Strengths and weaknesses in relation to other studies and comparison of results

This study contributes to the growing literature on medical student attitudes towards primary care, both in the UK [6, 13, 15, 18–20] and internationally [21–25]. It has one of the highest response rates (89%) and through the inclusion of open questions has generated a rich qualitative data set of over 1000 free text responses.

However, the cluster analysis failed to identify subgroups of individuals with similar career aspirations. This may be because students maintain broad career plans during medical school, however it is plausible that the data set is too small to identify any such groupings, should they exist. Indeed, a sample size of 280 is small compared to the larger UK and international studies. The reports supported by the Medical School Council – “By Choice, Not by Chance” [6] and “Destination GP” [15], included students from up to 30 medical schools, improving the generalisability of their findings within the country. However, these were not peer-reviewed studies.

Similarities between our study and others both in the UK and abroad suggest our results do not reflect just local issues. Tribalism and badmouthing of general practice is not unique to Oxford [6, 8, 15], and the low prestige of primary care compared to hospital specialities is not unique to the UK [19, 25]. In addition, the important role of GPs themselves as role models (both positive and negative) has been highlighted in numerous other studies [18, 20, 23]. The effect of peer-to-peer influence was highlighted by the recent “Destination GP” report: “91% of students believe that their peers hold negative views about general practice” and “35% of students indicated their peers at medical school as one of the most influential groups…” [15]. Our study did not appear to show such a strong effect of peer-to-peer influence. This may represent a real difference, but direct comparison is limited between questions with different constructs. Moreover, the above report also found that 51% of students believed that their peers held positive views about general practice, so peer influence may not necessarily be negative.

Our study considered the influence of three demographic variables: sex, course type (standard vs. graduate-entry) and school type. No effect of gender was found, in contrast to previous studies showing female medical students are more likely to favour general practice [13]. However, our study did not assess age, ethnicity, place of birth or seniority at medical school as factors influencing career choice. There is evidence from other studies that these variables may be influential, in particular that attitudes towards general practice change during medical school [13]. Our decision to combine results from final and penultimate year students (to produce a larger sample) meant that this study was unable to tease out any age effect.

### Meaning of the study: implications for clinicians and policymakers

Factors external to medical schools – such as the pressures on GPs and the low morale of the workforce – must be meaningfully addressed alongside any other initiative aiming to improve recruitment. For the factors internal to medical schools, this study reinforces the findings from other recently published reports, and supports many of the recommendations made.

Our study shows that community-based working is poorly valued by respondents. There is evidence from the UK of a positive correlation between the number of “authentic” (i.e. in a practice, with patient contact) sessions in general practice at medical school and progression to general practice [26]. Oxford falls into the lowest quarter for number of authentic sessions in general practice amongst UK medical schools [26]. Similarly, data from the US suggest that longer, more immersive community placements make students more likely to choose general practice [27]. We support the urgent call to re-evaluate funding systems in medical schools in order to provide longer, high quality community placements with motivated GPs who have the attitudes and personal qualities to be inspirational role models [6, 7].

The issue of tribalism splits opinion. Given the evidence that it impacts career choice [8], some have called for zero tolerance towards the badmouthing of general practice [28]. Others worry that “playing the denigration card risks GPs being perceived as victims”, which may be detrimental to the cause [29]. We support measures to increase awareness of, and resilience to, negative depictions of general practice by other specialties.

However, we also advocate positive action by GPs themselves. Our study has confirmed the central role GPs play in shaping medical student attitudes. As such, we suggest three approaches: greater involvement of GPs in clinical and non-clinical teaching throughout medical school, utilising GP trainees as “near peer” teachers and positive role models, and enhancing the profile of academic primary care within the teaching institution. At a university with a world leading department of academic primary care, it is alarming that some Oxford students believe that working in general practice limits research opportunities. Finally, like the recent Medical School Council reports [6, 15], we believe that supporting student-led GP societies will create a positive peer-to-peer effect and promote general practice as a dynamic, varied and stimulating career.

### Unanswered questions and future research

There is evidence from the UK and US that longer placements in general practice increase progression to this career choice [26, 27]. Future research should evaluate the effect of exposure to near-peer (GP trainees) and academic role models, as well as focusing on what makes a quality clinical placement in general practice.

## Conclusions

This study, from a single UK medical school, confirms and updates previous research from the UK and elsewhere that far fewer soon-to-graduate medical students are seriously considering general practice as a career than are needed for the future workforce. The majority of students neither value community-based working, nor view general practice as a prestigious career. Medical schools have a responsibility to address their formal curriculum (notably the short time which students spend in general practice compared with hospital specialties, and limited exposure to academic primary care and community-based research), but also the powerful “hidden curriculum”, including negative stereotyping by hospital specialists. Wider factors must also be addressed: exposure to GPs who are burnt out and disillusioned by the current pressures facing primary care has a profound and lasting negative effect. Clearly, improvements in GP recruitment require improvements in the working lives of GPs.

## Additional files

Additional file 1: **Appendix 1.** Questionnaire. Description: 19-item questionnaire, combining dichotomous, multiple choice, demographic and Likert response scale questions with free text questions (DOCX 15 kb)

Additional file 2: **Appendix 2.** Logistic regression analysis. Description: Logistic regression analysis to determine the best fitting explanatory variables for the attractiveness of a career in general practice. (DOCX 15 kb)

## Abbreviations

GP: Refers to a general practitioner or general practice
JR: Refers to the John Radcliffe Hospital, Oxford
UK: United Kingdom
US: United States
