# Supplementary material for: UK medical students’ attitudes towards their future careers and general practice: a cross-sectional survey and qualitative analysis of an Oxford cohort
Source: BMC Med Educ. 2018 Jul 4;18:160. doi: 10.1186/s12909-018-1197-z (PMC6030758; doi:10.1186/s12909-018-1197-z)
Supplement: Supplementary file 1 — Appendix 1. Questionnaire. Description: 19-item questionnaire, combining dichotomous, multiple choice, demographic and Likert response scale questions with free text questions (DOCX 15 kb) [file 12909_2018_1197_MOESM1_ESM.docx]

Questionnaire

1. Please indicate which career(s) if any you are currently considering
   1. Anaesthesia/intensive care
   2. Emergency medicine
   3. General practice
   4. Medical specialties
   5. Obstetrics and gynaecology
   6. Oncology
   7. Pathology
   8. Paediatrics
   9. Psychiatry
   10. Public health
   11. Radiology
   12. Surgical specialties
   13. Non-clinical medicine
   14. Career outside of medicine
   15. Not applicable
2. If you have given a potential career option in Q1 please indicate when you first started considering this
   1. School
   2. Gap year
   3. Pre-clinical years
   4. During clinical course
   5. Not sure
   6. Other please state below
   7. Not applicable
3. Are you considering or did you apply for an Academic Foundation Post?
   1. Yes
   2. No
4. To what extent do you agree or disagree that these factors are important when thinking about your career choices [Likert scale: strongly agree, agree, uncertain, disagree, strongly disagree]?
   1. Job satisfaction
   2. Professional status
   3. Length of training
   4. Research opportunities
   5. Career development
   6. Teamwork
   7. Community-based working
   8. Reasonable working hours
   9. Close relationship with patients
   10. Income potential
   11. Flexibility in location
5. Please state any other factors relevant to your career choice [free text]
6. Regarding all the factors you have stated as influencing your career choice, please state which you think is the most important [free text]
7. How do you view primary care as a potential career choice for you currently [Likert scale: very attractive, attractive, neutral, unattractive, very unattractive]?
8. Please briefly explain your views towards primary care as a potential career choice [free text]
9. Thinking more about your views regarding primary care, please respond to the following statements. Compared to other careers, a career in primary care is likely to offer me [Likert scale: strongly agree, agree, uncertain, disagree, strongly disagree]
   1. Job satisfaction
   2. Professional status
   3. Research opportunities
   4. Career development
   5. Teamwork
   6. Reasonable working hours
   7. Close relationship with patients
   8. Higher income
10. Regardless of your current career plans, which of the following has shaped or influenced your views towards primary care as a career in either a positive or negative way [Likert scale: strongly positive, positive, neutral, negative, strongly negative]?
    1. Year 5 GP placement
    2. Academic GPs
    3. Hospital consultants
    4. Hospital junior doctors
    5. College tutors
    6. Other medical students
    7. Members of your family
    8. Overall culture of Oxford medical school
    9. Your current or previous GP
    10. Medical media
    11. Non-medical media
    12. Current medico-political climate
11. Has anyone else influence your views towards primary care as a career option? If so please say who and why [free text]
12. Are there any individuals who have (directly or indirectly) particularly encouraged you to consider primary care as a career option? If so please state their role (e.g. orthopaedic surgeon) and how they have encouraged you [free text]
13. Are there any individuals who have (directly or indirectly) particularly discouraged you from considering primary care as a career option? If so please state their role (e.g. GP) and how they have discouraged you. [free text]
14. In general terms how do you think the professional status of general practitioners compares with that of hospital specialists?
    1. Don’t know
    2. Higher status
    3. About the same status
    4. Lower status
15. Concerning your secondary school education, please indicate which of the following best applies to you
    1. Non-selective state school in UK
    2. Selective state school in UK
    3. Private school in UK
    4. Secondary education outside UK
    5. Other
16. Are you
    1. Female
    2. Male
    3. Rather not say
17. Are you on the
    1. Standard medical course
    2. Graduate-entry medical course
18. Are you considering working abroad after
    1. No
    2. Yes (please give brief details)
19. Finally thank you for your responses. Regardless of your current career plans it would be much appreciated if you could briefly respond to the following statement: I would be more likely to consider a career in primary care if...
