# Supplementary material for: UK medical students’ attitudes towards their future careers and general practice: a cross-sectional survey and qualitative analysis of an Oxford cohort
Source: BMC Med Educ. 2018 Jul 4;18:160. doi: 10.1186/s12909-018-1197-z (PMC6030758; doi:10.1186/s12909-018-1197-z)
Supplement: Supplementary file 2 — Appendix 2. Logistic regression analysis. Description: Logistic regression analysis to determine the best fitting explanatory variables for the attractiveness of a career in general practice. (DOCX 15 kb) [file 12909_2018_1197_MOESM2_ESM.docx]

Logistic regression analysis in SPSS: We modelled the association between the attractiveness of a career in general practice (attractive or very attractive = 1) and a list of importance factors (Q4) and influencing factors (Q10). We performed separate models for each, one for influencing and one for importance factors. The table below shows the results of the final model in which those variables regarded to be positively associated with the attractiveness of a career in general practice were combined in a stepwise approach.

|  | Adjusted Odds Ratio ^a^ | 95% Confidence Interval Lower Limit | 95% Confidence Interval Upper Limit | p-value |
| --- | --- | --- | --- | --- |
| Year 5 GP placement | 9.636 | 3.972 | 23.379 | <0.001 |
| Community based working | 6.622 | 3.004 | 14.595 | <0.001 |
| Reasonable working hours | 3.274 | 1.481 | 7.238 | 0.003 |
| Current medico-political climate | 2.019 | 0.999 | 4.079 | 0.05^a^ |
| Constant | 0.024 |  |  | <0.001 |

1. We used the default parameters in SPSS as part of the stepwise selection process. This means that to enter the model a variable must have a significant “unadjusted” OR, that is a p-value less or equal to “a” 0.05. To exclude a variable once it is in the model the “adjusted” OR level is less stringent, with exclusion for variables with p-values greater than or equal to 0.1. Therefore a p value of 0.05 is significant in this final, single model.
